# Nanoparticles for Tendon Healing and Regeneration: Literature Review

**DOI:** 10.3389/fnagi.2016.00202

**Published:** 2016-08-22

**Authors:** Paolo D. Parchi, Orazio Vittorio, Lorenzo Andreani, Pietro Battistini, Nicola Piolanti, Stefano Marchetti, Andrea Poggetti, Michele Lisanti

**Affiliations:** ^1^First Orthopaedic Division, Department of Translational Research and New Technology in Medicine and Surgery, University of PisaPisa, Italy; ^2^Lowy Cancer Research Centre, Children's Cancer Institute Australia, UNSW AustraliaSydney, NSW, Australia; ^3^Australian Centre for NanoMedicine, UNSW AustraliaSydney, NSW, Australia

**Keywords:** nanoparticles, tendon injuries, scaffold, silver nanoparticles, gold nanoparticles

## Abstract

Tendon injuries are commonly met in the emergency department. Unfortunately, tendon tissue has limited regeneration potential and usually the consequent formation of scar tissue causes inferior mechanical properties. Nanoparticles could be used in different way to improve tendon healing and regeneration, ranging from scaffolds manufacturing (increasing the strength and endurance or anti-adhesions, anti-microbial, and anti-inflammatory properties) to gene therapy. This paper aims to summarize the most relevant studies showing the potential application of nanoparticles for tendon tissue regeneration.

## Introduction

Tendon injuries could be caused by trauma, but most of them are the result of gradual wear and tear of the tendon from overuse or aging (Thomopoulos et al., 2015). A tendon injury may seem to happen suddenly, but usually it is the result of many tiny tears to the tendon that have happened over time (Molloy and Wood, 2009). Tendons show limited regeneration potential and, most of the time there is scar tissue formation which causes inferior mechanical properties (Molloy and Wood, 2009). Clinically, the healing of Achilles tendon usually takes 4–8 weeks; however, a full return to sport activities is only recommended after a long span of 4–12 months.

To overcome these problems new strategies based on stem cell transplantation and growth factors have been proposed (Sahni et al., 2014; Lui, 2015). The ability of several growth factors to improve the healing response and decrease scar formation is described in different preclinical studies (Sahni et al., 2014; Lui, 2015). Besides the application of growth factors and stem cell transplantation, innovative research focuses on the development of nano-structured scaffolds to improve the healing response in tendon injuries (Oragui et al., 2012).

Structurally tendons and associated extracellular matrices are composed of nanostructured materials. For this reason, in the last years there was a growing interest to develop novel nano-materials for tendon regeneration. Nanotechnology is the precise placement, measurement, manipulation, and modeling of matter that consists of 4–400 atoms. The range below 100 nm is important because the classic law of physics change, resulting in novel physical properties that allow researchers to produce new materials with exact properties, such as size and strength beyond conventional limits. Nanomaterials have been proposed to improve tendon regeneration and to decrease the form of scar tissue and fibrous adhesions.

Nanoparticles (NPs) are a material (0-D), in which almost a dimension is < 100 nm. NPs represent a bridge between the material of conventional size and structures at the atomic level. Indeed, biomedical engineering studied for decades the important characteristics (size, magnetizability, functionalization) to be exploited to create new treatment strategies in different medical areas (oncology, nerve regeneration and tissue regeneration, infettivology, radiology, etc.).

The aim of this paper is to review and discuss the current advances in nanoparticles applications for tendon tissue regeneration.

The nanoparticles could be exploited in several ways: in the manufacture of scaffolds, increasing the strength, and endurance or anti-adhesions, anti-microbial, and anti-inflammatory properties; as a carrier in gene therapy, for an anti-adhesions and anti-inflammatory activity; using directly their anti-inflammatory properties and relationship with the extracellular matrix (ECM) and cell surfaces; aid in the iontophoresis and phonophoresis using anti-inflammatory properties; labeling and tracking stem-cells with MRI.

## Nanoparticles for direct medical applications

An important application of nanoparticles is represented by their use as nano-delivery system for the treatment of tendinitis. In particular, nanoparticles have been used in Iontophoresis and phonophoresis, techniques capable of enhancing drug penetration through the skin. Phonophoresis uses ultrasound waves to deliver drugs through the skin, and iontophoresis uses low level electric current. Both techniques are usually used to treat inflammatory conditions such as tendonitis. Dohenert et al. studied the possibility to improve the drug transport during Iontophoresis and phonophoresis using gold nanoparticles (GNPs) as drug nanotrasporter (Dohnert et al., 2012, 2015). They studied the use of GNPs functionalized with Diclofenac diethylammonium in the treatment of a tendinopathy in animal model. The results of this study showed that the use of GNPs is associated with a strong a down-modulation of the inflammatory response (reduction IL-1β and TNF-α) which resulted by an improved of drug delivery to the site of injury (Dohnert et al., 2012, 2015). The authors underline that GNPs exerted anti-inflammatory and synergistic action: enabling the transport of the drug used and enhancing the therapeutic role of iontophoresis and phonophoresis (Dohnert et al., 2012, 2015).

## Nanoparticles for microRNA delivery

The delivery of nucleic acid *in vivo* has been preferentially performed by using viral vectors with some concerns about the safety of these procedures in patients (Vannucci et al., 2013). Nanotechnology has developed several nanoparticles to be used for gene therapy to replace the viral vectors and avoid their side effects (Raffa et al., 2011). Zhou et al. evaluated the use of nanoparticles as non-viral vector for gene therapy to prevent peritendineus adhesion formation (Zhou et al., 2013). The authors reported that the miRNAs reducing the expression of TGF-b1 (induce fibrotic changes and adhesion formations in the tissues like tendons) were inserted into the plasmid, and then the generated plasmids were loaded into PEI-polylactic-co-glycolic acid (PLGA) nanoparticles for preventing peritendinous adhesion (Zhou et al., 2013). Interestingly, they performed their study *in vitro*, using a culture of primary tenocites from flexor digitorum profundus tendon of Leghorn chicken, and *in vivo* by using the same type of tendon. The analysis of the data obtained by using electron microscopy imaging, molecular biology techniques, and biomechanical tests, underline the potential of PLGA nanoparticles as an innovative and efficient agent for gene delivery in tendon. In fact, the transfection of miRNA by PLGA nanoparticles resulted in an inhibition of TGF-b1 expressions, which in turn induced the repair of the tendon (Zhou et al., 2013). The results of this study showed the strength of the treated tendons was lower of than in the control group due to the down regulation of cells migration, proliferation, adherence, apoptosis, and of the secretion of ECM related to the inhibition og TGF-b1 (Zhou et al., 2013). The authors concluded that the purely inhibition of the expression of TGF could not achieve the desired healing effect of injured tendon. It was hyphosesized that a better results on tendon repair could be achieved by combining TGF-b1 miRNA plasmid with other miRNA for other growth factor genes, to be delivered simultaneously by nanoparticles (Zhou et al., 2013).

Brevet et al. (2014) performed functionalization of Mesoporous silica nanoparticles (MSN) with L-histidine and show a better efficiency of histidine-functionalized MSN in transfecting cells than imidazole- or amino- functionalized MSN (Brevet et al., 2014); the study was conducted *in vitro* and *in vivo* (Mice Achilles tendon; Brevet et al., 2014). The results confirmed a good gene delivery efficiency *in vitro*, but it was lower *in vivo* (Brevet et al., 2014). However, additional studies are working in the modification of MSN to increase their potential as delivery system for nucleic acid for the treatment of tendon injuries.

## Nanoparticles for scaffold manufacturing

Biological or synthetic scaffolds has been introduced to give a mechanical support to the tendon during the healing process (Longo et al., 2012). In regenerative medicine scaffolds are often used in combination with growth factors and stem cells to have a structural (mechanical) and biological support to the tissue healing. In tissue engineering the scaffolds could also functionalization using nanoparticles to give them new chemical and physical properties.

Karthikeyan et al. (2011) developed a system to produce functional biofibers containing silk fibers (SF) coated with chitosan and impregnated with silver nanoparticles (Ag–C–SF). Chitosan [poly-b-(1-4)-D-glucosamine] is a sustainable, biocompatible, biodegradable, and antimicrobial polysaccharide of great relevance in many fields of application. They tested the fibers with microbiological assays, Scanning electron microscopy (SEM), infrared spectroscopy, AFM studies and thermo gravimetric analysis (Karthikeyan et al., 2011). The results shown antimicrobial activity (nanoparticles enter into bacteria, inhibit the ATP Synthesis and denature DNA, and blocking the respiratory chain) and increased thermal stability (Karthikeyan et al., 2011). The authors emphasize that this fiber might be a promising material in wound healing and tendon reconstruction (Karthikeyan et al., 2011).

Liu et al. (2013a) studied the behavior of silver nanoparticles (AgNPs) directly electro spun into biodegradable poly (L-lactide; PLLA) fibrous membrane (Liu et al., 2013a). Electro spun fibrous membranes are attractive barriers for tissue separation and drug delivery to get drug-loaded materials with lengthened releasing time because of their large surface area and controlled porous structure. The transmission electron microscopy (TEM) micrographs of the fibers showed that the AgNPs were successfully electro spun into the PLLA fibers in different contents with ability of Ag ion release (Liu et al., 2013a). The anti-proliferation effect of the AgNP-loaded PLLA fibrous membranes was observed on fibroblasts. Furthermore, no cytotoxicity was detected (Liu et al., 2013a). The broad-spectrum topical antimicrobial activity of AgNP-loaded PLLA fibrous membranes on *S. epidermidis, S. aureus*, and *P. aeruginosa* was certificated (Liu et al., 2013a). These properties make silver ions just suitable for prompting anti-adhesion treatment and simultaneously early prevention of infection. Moreover, although cell proliferation on the surfaces of AgNP-loaded PLLA fibrous membranes was worse than on the surface of PLLA fibrous membrane, the traditional negative effect that prevents cell proliferation was now treated as the positive effect that inhibits adhesion formation (Liu et al., 2013a). In fact, the prevention of bacterial adhesion should assist in reduction of device associated infection. This *in vitro* study showed that the AgNP-loaded PLLA fibrous membranes have a significant effect of preventing cell adhesion and proliferation without significant cytotoxicity (Liu et al., 2013a).

Chen et al. (2014) also used an electro spun fibrous membrane loaded with AgNPs (Chen et al., 2014). They prepared a combination of Ibuprofen (IBU) and Ag for decreasing kidney and liver damages caused by high dose of Ag, while maintaining good anti-adhesion effect (Chen et al., 2014). Also in this *in vitro* study, it was demonstrated that the electro spun Ag/IBU-loaded PLLA fibrous membrane not only prevented cell adhesion and proliferation but also reduced bacterial infection through its stable release of silver ions and IBU (Chen et al., 2014).

In another study of Liu et al. (2013b), PLLA was loaded with growth factors (heparin binding site of basic fibroblast growth factor [bFGFs]; Liu et al., 2013b). bFGF has been shown to stimulate angiogenesis, cellular differentiation, migration, proliferation, and matrix synthesis *in vivo* and *in vitro* in a variety of tendons. Growth factors could be used to promote tendon cells differentiation but one of the main limits relate to their use in the clinical practice are due to their poor biodisponibility *in vivo*. The possible way to overcome this problem is to use the growth factors in combination with scaffolds.

In this study, one preformulated dextran glassy nanoparticles (DGNs) loaded with bFGF were electro spun into a PLLA copolymer fiber to secure the biological activity of bFGF in a sustained manner and thus to enhance tendon healing and simultaneously prevent peritendinous adhesion (Liu et al., 2013b). Their analysis of data *in vitro* and *in vivo* (Achilles tendon of Spague-Dawley rat) shows a good protein encapsulation efficiency of the bFGF/DGNs-PLLA membrane with a release kinetic of nearly 30 days; the bFGF/ DGNs-loaded PLLA fibrous membrane can release bFGF sustainably and secure the bioactivity of bFGF (Liu et al., 2013b). The authors concluded that bFGF/DGNs-loaded PLLA membrane can protect the bioactivity of bFGF in a sustained manner for promotion of tendon healing and simultaneous adhesion prevention (Liu et al., 2013b).

Recently, there was a growing interest regarding the use of Nano cellulose materials for scaffolds design. He et al. (2014) used cellulose Nano crystals (CNCs) as reinforcement polymer Nano composites (He et al., 2014). The authors evaluated the possibility of fabricating uniaxially aligned electro spun nanofiber nonwovens from cotton cellulose and their potential application in tissue engineering (He et al., 2014). Morphology investigation from SEM images indicated that most of the obtained cellulose nanofibers were aligned and a more uniform morphology can be obtained with the incorporation of CNCs. Cell culture experiments demonstrated that the electro spun cellulose reinforced with CNCs promote fibroblast cells attachment and proliferation in the entire scaffold (He et al., 2014).

## Nanoparticles for tendon healing (anti-microbial effect, anti-adhesion effect and extracellular matrix composition modulation)

Silver nanoparticles have also been recognized as antimicrobial agents because they inhibit ATP Synthesis in the microorganism, denature the DNA and block the respiratory chain (Klueh et al., 2000; Kumar et al., 2005; Morones et al., 2005). AgNPs not only exert anti-microbial effect, but are also capable of accelerating burn wound healing due their antiflogistic effects (Klueh et al., 2000; Kumar et al., 2005; Morones et al., 2005).

Kwan et al. (2014) investigated the effects of silver nanoparticles in the tendon healing process *in vitro* and *in vivo* using Achilles Sprague Dawnley rat (Kwan et al., 2014). In the *in vitro* evaluation the authors showed that the silver nanoparticles promote the proliferation of primary tenocytes to AgNPs and the production of ECM components (Kwan et al., 2014). In the *in vivo* evaluation the tensile tests showed that the tensile modulus of the nanoparticles-treated group was significantly better in comparison to the control group but a quite less than the normal tendon (Kwan et al., 2014). The silver nanoparticles accelerated the tendon healing process and modulate the ECM composition (more and better quality collagen fibrils). The results of this study showed that silver nanoparticles have a positive effect in Achilles tendon healing, through boosting cell proliferation and stimulating the production of collagen and proteoglycans (Kwan et al., 2014). The silver nanoparticles also showed an antiflogistic effect reducing the formation of scar tissue and adhesions (Kwan et al., 2014).

Empson et al. (2014) explored the *in vitro* biomechanical and cellular response of the therapy with nanoparticles for damaged connective tissues (Empson et al., 2014). They hypothesized that the controlled and localized injection of biocompatible NPs into damaged connective tissues would enhance matrix mechanical properties, evidenced by increased stiffness, and yield strength (Empson et al., 2014). The effects of NPs, namely, single-walled CNHs and CNCs, on damaged connective tissue mechanical properties were examined. In the study conducted by Empson et al. the authors evaluated the effects of the use of nanoparticles CNHs and CNCs using different types of connective tissues the porcine skin, that mimics the mechanical and biological properties of human ligaments and tendons, and the porcine tendon as a model of the treatment of target tissues (Empson et al., 2014). They analyzed data with atomic force microscopy (ATM), dynamic light scattering (DLM), cell cultures and mechanical tests. Authors underline that the results presented in this study show the feasibility of using CNHs and CNCs to locally reinforce damaged connective tissues (Empson et al., 2014).

Recent works have also shown the ability of nanoparticles (NPs) to modulate the cellular responses (Fisher et al., 2010; Kwan et al., 2011) and the ECM mechanical properties (Bhattacharyya et al., 2006; Li et al., 2009).

In addition to triggering a cellular response, NPs have also been shown to enhance the mechanical properties of natural polymer matrices; examples include chitosan, regenerated cellulose, and decellularized porcine diaphragm tendon (Qi et al., 2009; Deeken et al., 2011).

## Nanoparticles and magnetic resonance imaging (MRI)

Nanoparticles can be used to improve the quality of MR imaging. Yang et al. (2013) studied the feasibility of labeling tendon stem cells (TSCs) with super-paramagnetic iron oxide (SPIO) nanoparticles to track TSCs *in vivo* using MRI (Yang et al., 2013). Although bone marrow mesenchymal stem cells have been used to repair injured tendons, they frequently cause bone formation in healed tendons. Therefore, TSCs may be more suitable than BMSCs to effectively repair acute and chronic tendon or ligament injuries.

The authors conducted an *in vitro in vivo* (rabbit model) study in which the TSCs has been incubated with appropriate concentration of SPIO (Yang et al., 2013). The results of this study showed that the TSCs maintain their biological characteristics and they could be traceable by MRI. The mechanism of SPIO cellular uptake is likely through receptor-mediated endocytosis and therefore maintaining the cells endocytosis capabilities is very important (Yang et al., 2013). In general, higher the SPIO dosag, longer labeling efficiency will be maintained. However, high doses of SPIO may have detrimental effects on cells, such as reduction in cell viability. Therefore, the balance between labeling efficiency, cell viability, and MRI signal should be considered when labeling cells. Comparing the results from using 100, 50, and 25 lg Fe/mL of SPIO, they found that high labeling efficiency without causing apparent changes to cell morphology can be achieved at 50 lg Fe/mL of SPIO concentrations (Yang et al., 2013). The authors underline that one limitation of this study is that MRI does not have sufficient resolution to detect individual cells and differentiate the “status” of labeled cells (Yang et al., 2013). The authors concluded that SPIO labeling did not change TSC viability, proliferation, or stemness, and that labeled cells could be tracked by MRI in a rabbit tendon injury model (Yang et al., 2013).

## Conclusion

Structurally tendons and associated extracellular matrices are composed of nanostructured materials. For this reason, in the last years there was a growing interest on new approaches for tendon regeneration based on nano-materials. Nanoparticles (NPs) in which almost a dimension is < 100 nm, represent a bridge between the conventional size materials, that are actually used in orthopedic surgery, and the atomic level tendon structures. In this review, we have presented some of the papers describing how nanoparticles could play an important role in tendon healing: labeling TSCs, working as carrier for gene therapy and for drug delivery, allowing the fabrication of a new generation of bioactive scaffolds and modulating the cellular and the ECM response. An important point to be addressed before the translation of nanomaterials to the clinic, is to assess the safety of these nanoparticles in the human body when they are used alone or as a component of an implantable devices.

It is clear that more work has to be done to find the best approach for tendons regeneration. Nanotechnology has a big potential in this field. This has become a multidisciplinary challenge and we believe that collaboration between orthopedic surgeons and experts in nanotechnology can make the difference in this challenge.

## Author contributions

PP: online research and abstract selection, coordination between authors and general paper composition, Abstract, Introduction, manuscript revision process and manuscript preprint editing. OV: online research and abstract selection, coordination between authors and general paper composition, Conclusion. LA: Nanoparticles for direct medical applications. PB: Nanoparticles for microRNA delivery. NP: Nanoparticles for scaffold manufacturing. SM: Nanoparticles and extracellular matrix (ECM) and cellular surface modulation. AP: Nanoparticles and magnetic resonance imaging (MRI). ML: Supervision.

### Conflict of interest statement

The authors declare that the research was conducted in the absence of any commercial or financial relationships that could be construed as a potential conflict of interest.
